# Androgen Receptor Splice Variant 7 Drives the Growth of Castration Resistant Prostate Cancer without Being Involved in the Efficacy of Taxane Chemotherapy

**DOI:** 10.3390/jcm7110444

**Published:** 2018-11-16

**Authors:** Yasuomi Shimizu, Satoshi Tamada, Minoru Kato, Yukiyoshi Hirayama, Yuji Takeyama, Taro Iguchi, Marianne D. Sadar, Tatsuya Nakatani

**Affiliations:** 1 Department of Urology, Graduate School of Medicine, Osaka City University, Osaka 545-8585, Japan; yasuomis0922@gmail.com (Y.S.); s-tamada@med.osaka-cu.ac.jp (S.T.); yuji101@outlook.jp (Y.T.); taro@msic.med.osaka-cu.ac.jp (T.I.); nakatani@med.osaka-cu.ac.jp (T.N.); 2 Genome Sciences Centre, BC Cancer, Vancouver, BC V5Z 1L3, Canada; ukiyoc@hotmail.com (Y.H.); msadar@bcgsc.ca (M.D.S.)

**Keywords:** androgen receptor, docetaxel, cabazitaxel, castration-resistant prostate cancer, chemotherapy, P-glycoprotein, EPI-002, splice variant

## Abstract

Expression of androgen receptor (AR) splice variant 7 (AR-V7) has been identified as the mechanism associated with the development of castration-resistant prostate cancer (CRPC). However, a potential link between AR-V7 expression and resistance to taxanes, such as docetaxel or cabazitaxel, has not been unequivocally demonstrated. To address this, we used LNCaP95-DR cells, which express AR-V7 and exhibit resistance to enzalutamide and docetaxel. Interestingly, LNCaP95-DR cells showed cross-resistance to cabazitaxel. Furthermore, these cells had increased levels of P-glycoprotein (P-gp) and their sensitivity to both docetaxel and cabazitaxel was restored through treatment with tariquidar, a P-gp antagonist. Results generated demonstrated that P-gp mediated cross-resistance between docetaxel and cabazitaxel. Although the LNCaP95-DR cells had increased expression of AR-V7 and its target genes (UBE2C, CDC20), the knockdown of AR-V7 did not restore sensitivity to docetaxel or cabazitaxel. However, despite resistance to docetaxel and carbazitaxel, EPI-002, an antagonist of the AR amino-terminal domain (NTD), had an inhibitory effect on the proliferation of LNCaP95-DR cells, which was similar to that achieved with the parental LNCaP95 cells. On the other hand, enzalutamide had no effect on the proliferation of either cell line. In conclusion, our results suggested that EPI-002 may be an option for the treatment of AR-V7-driven CRPC, which is resistant to taxanes.

## 1. Introduction

The primary effective treatment for most recurring prostate cancer (PC) is androgen deprivation therapy (ADT). Although initially effective, the malignancy will eventually form castration-resistant prostate cancer (CRPC) [1]. Current treatment options for CRPC are androgen receptor- (AR-) targeted therapies, such as enzalutamide and abiraterone, as well as taxanes, such as docetaxel and cabazitaxel. However, no curative CRPC therapy is available for the presentation of treatment resistance [2]. The mechanisms of CRPC development include overexpression of AR [3,4], gain-of-function mutations in the AR ligand-binding domain (LBD) [5], intratumoral androgen synthesis [6], altered expression and function of the AR coactivators [7,8], aberrant post-translational modification of the AR [9], and the AR splice variants (AR-Vs) lacking the LBD [10]. Expression of AR-V7 in human prostate cancer cell lines mediates resistance to enzalutamide and abiraterone [11,12]. EPI compounds are AR amino-terminal domain- (NTD-) targeting drugs that block the transcriptional activities of full-length (FL)-AR and AR-Vs in vitro and exhibit antitumor activity in CRPC xenografts [13,14,15]. Resistance to taxane-based chemotherapy is frequently attributed to the overexpression of the transporter protein, P-glycoprotein (P-gp), which is also known as ATP-binding cassette subfamily B member 1 (ABCB1) or multidrug resistance protein 1 (MDR-1) [16,17]. Prostate cancer specimens from CRPC patients have increased levels of P-gp [18]. Cabazitaxel is highly cytotoxic with a low affinity for P-gp [19], and therefore, it is not considered to be clinically cross-resistant with docetaxel, thereby providing a survival benefit for docetaxel-pretreated patients [20]. However, a recent report indicated that P-gp could mediate cabazitaxel–docetaxel cross-resistance in advanced prostate cancer [21]. Alternative mechanisms of resistance to taxanes may involve tubulin mutations [22], although the precise association has yet to be elucidated. The presence of AR-V7 in circulating tumor cells from men with metastatic CRPC is not associated with primary resistance to taxane chemotherapy [23,24], which is contrary to pre-clinical data suggesting that the expression of AR-V7 mediates resistance to docetaxel in LuCap23.1 human prostate cancer xenografts [25]. In this study, we focused specifically on the role of AR-V7 in taxane resistance in pre-clinical models of prostate cancer to address this clinically important question. We employed the CRPC cell line, LNCaP95, which endogenously expresses AR-V7, to examine the status of cross-resistance between docetaxel and cabazitaxel, and to assess the involvement of AR-V7 with taxane resistance. We further evaluated the effect of EPI-002, an NTD-targeting drug, on enzalutamide resistant LNCaP95 cells with acquired resistance to taxanes.

## 2. Materials and Methods

### 2.1. Cell Lines and Culture Conditions

Prostate cancer cell lines, DU145, PC3, and LNCaP were purchased from the American Type Culture Collection (ATCC). LNCaP95 was a generous gift from Dr. Jun O. Luo (Johns Hopkins University, Baltimore, MD, USA). Cells were authenticated by short tandem repeat analysis (Takara Bio Inc., Shiga, Japan) and then tested by DDC Medical (Thermo Fisher Scientific, Waltham, MA, USA) in April 2018, to ensure that the cells were mycoplasma-free. Cells were maintained as monolayer cultures at 37 °C and 5% CO_2_. The cell line DU145 was cultured in DMEM supplemented with 10% FBS, PC-3 in RPMI 1640 with 10% FBS, LNCaP in phenol red-free RPMI 1640 with 10% FBS, and LNCaP95 in phenol red-free RPMI 1640 with 10% charcoal stripped serum (CSS). A docetaxel-resistant cell line variant, LNCaP95-DR, was developed over a period of 6 months by exposure to gradually increased concentrations of docetaxel (Sigma-Aldrich, St. Louis, MO, USA). A time-matched parental cell line, LNCaP95-C, was developed in a medium containing vehicle (DMSO) at the corresponding concentration. Finally, LNCaP95-DR cells were maintained in medium containing 15 nM docetaxel.

### 2.2. Cell Proliferation Assay

The effect of drugs on cell proliferation was assessed using the Premix WST-1 Cell Proliferation Assay System (Takara Bio) according to the manufacturer’s protocol. Cell viability was normalized to the viability of vehicle-treated control cells (DMSO). 

BrdU ELISA was performed to evaluate the inhibitory effect of EPI-002 on the proliferation of cells. LNCaP95-P, LNCaP95-C, and LNCaP95-DR cells were treated with vehicle (DMSO) or EPI-002 for 48 h, and BrdU incorporation was measured using the BrdU ELISA kit (Roche Diagnostics, Basel, Switzerland).

### 2.3. Western Blot Analysis

Western blots were performed as previously described in Reference [26]. The primary antibodies used were: AR (1:1000; Santa Cruz Biotechnology), AR-V7 (1:400; Precision), GR (1:1000; BD transduction laboratories), PSA (1:1000; Santa Cruz Biotechnology), FKBP5 (1:1000; Santa Cruz Biotechnology), UBE2C (1:1000; Boston Biochem), NSE (1:1000; Merck), Mdr-1 (1:1000; Santa Cruz), Aurora A (1:1000), BRN-2 (1:1000), total-STAT3 (1:1000), p-STAT3Tyr705 (1:1000), total-AKT (1:1000), p-AktSer473 (1:1000), total-S6 (1:1000), p-S6 (1:2500), total-p44/42MAPKErk1/2 (1:1000), p-p44/42MAPKErk1/2 (1:1000), 110α (1:1000), 110β (1:1000), 110γ (1:1000), PI3KClass III; (1:1000), p85 (1:1000), 4EBP1 (1:1000), and p-4EBP1 (1:1000), from Cell Signaling Technology. Beta-actin (1:1000, Abcam and Cell Signaling Technology) was used as a loading control.

### 2.4. Real-Time Quantitative Reverse Transcription PCR (Real-Time RT-qPCR)

Total RNA was isolated using the RNAqueous Total RNA Isolation Kit (Life Technologies, Waltham, MA, USA) and it was reverse transcribed to cDNA using the High Capacity cDNA Reverse Transcription Kit (Applied Biosystems, Waltham, MA, USA). Real-time RT-qPCR was performed in triplicate for each biological sample. Transcript levels for each gene were normalized to levels of the GAPDH transcript. Primers were purchased from Applied Biosystems: AR (Hs00171172_m1), KLK3 (Hs02576345_m1), FKBP5 (Hs01561006_m1), UBE2C (Hs00964100_g1), CDC20 (Hs00426680_mH), GAPDH (Hs00266705_g1), AR-V7 (forward, 5′-CCATCTTGTCGTCTTCGGAAATGTTA-3′; reverse, 5′-TTTGAATGAGGCAAGTCAGCCTTTCT-3′).

### 2.5. AR-Driven PSA(6.1kb)-Luciferase Reporter Gene Assay

PSA (6.1kb)-luciferase reporter plasmid encodes nucleotides −6000/+12 relative to the transcription start site of the human PSA/KLK3 gene and it includes the PSA promoter, with AREII (−395 to 376) and AREI (−170 to −156), and enhancer regions with AREIII (−4148 to −4134), as described in References [27,28]. LNCaP95-C and LNCaP95-DR cells seeded in 24-well plates were transfected using FuGENE HD Transfection Reagent (Promega, Madison, WI, USA), with a plasmid encoding the prostate-specific antigen (PSA) (6.1 kb)-luciferase reporter gene construct. The next day, the cells were pre-treated with vehicle (DMSO), enzalutamide (10 µM), docetaxel (5 nM), or cabazitaxel (10 nM) for 1 h before adding R1881 or EtOH (vehicle) under serum-free, phenol red-free conditions. After 48 h of incubation, the cells were harvested and lysed using the lysis buffer that was provided with the Luciferase Assay System (Promega). PSA-luciferase activity was measured using with the Wallac 1420 ARVOsx multi-label plate reader (PerkinElmer, Waltham, MA, USA) and normalized to protein concentration by the Bradford method as explained in Reference [29].

### 2.6. Knockdown of AR-V7

AR-V7 expression was transiently knocked down in LNCaP95-DR cells using Lipofectamine^TM^ RNAiMAX (Invitrogen, Carlsbad, CA, USA) according to the manufacturer’s instructions. AR-V7 siRNAs (Silencer^®^ Select siRNAs) were obtained from Life Technologies^TM^ (Grand Island, NY, USA). The sense sequence of siRNA for AR-V7 was 5′-GUAGUUGUGAGUAUCAUGATT-3′.

### 2.7. Statistical Analysis

Statistical analyses were performed using GraphPad Prism 7 (GraphPad Software, Inc., La Jolla CA, USA). All experiments were performed in triplicates for each biological sample. Data for cell proliferation assays, real-time RT-qPCR, and luciferase assays were depicted as mean ± SD from 3 to 4 independent experiments. IC50 values and 95% confidence intervals (CI) were calculated using the nonlinear regression analysis of percentage inhibition. The comparison of LogIC50 was calculated using the extra sum-of-squares F-test. One-way ANOVA followed by a Sidak’s post hoc test was used to assess the difference between the data of real-time RT-qPCR and luciferase assay. *p* < 0.05 was considered to indicate a statistically significant difference.

## 3. Results

### 3.1. LNCaP95-DR Cells Were Cross-Resistant to Cabazitaxel

**Figure 1:**
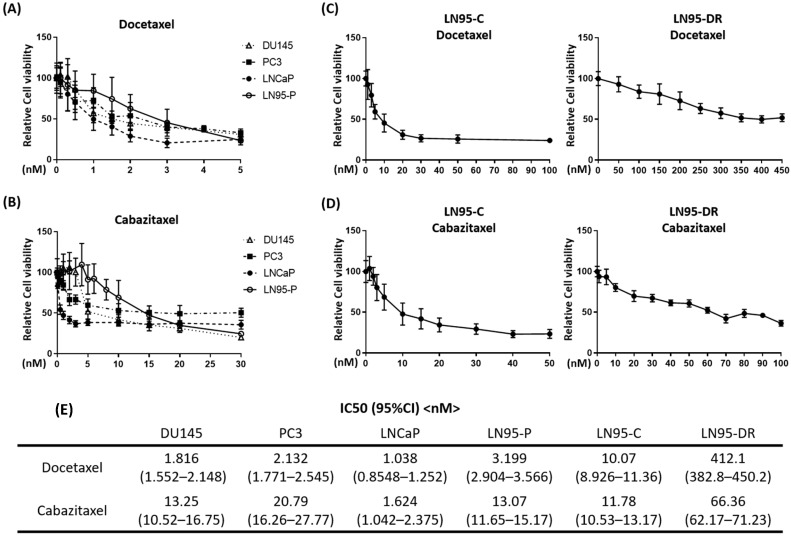
LN95-DR shows cross-resistance to cabazitaxel. Dose responses for docetaxel (**A**) and cabazitaxel (**B**) on the viability of prostate cancer cell lines (DU145, PC3, LNCaP, and LN95-P) assessed by the MTT assay; Dose responses for docetaxel (**C**) or cabazitaxel (**D**) on the viability of LN95-C and LN95-DR after 72 h; (**E**) A table showing IC50 values and 95% confidence intervals for docetaxel and cabazitaxel on prostate cancer cell lines. LN95-P: parental LNCaP95; LN95-C: time-matched parental LNCaP95 cells treated with DMSO as a vehicle control; LN95-DR: LNCaP95 with acquired resistance to docetaxel.

To evaluate the inhibitory effect of docetaxel and cabazitaxel on prostate cancer cell lines, the MTT assay was performed (Figure 1A,B). LNCaP cells were highly sensitive to docetaxel and cabazitaxel, whereas LNCaP95 cells were less sensitive than LNCaP cells. A docetaxel resistant LNCaP95 cell line, LNCaP95-DR, was obtained by exposing parental cells to gradually increasing concentrations of docetaxel. As shown in Figure 1C, LNCaP95-DR cells were significantly less sensitive to docetaxel than LNCaP95-C cells. Furthermore, LNCaP95-DR cells were less sensitive to cabazitaxel than LNCaP95-C cells (Figure 1D). A table showing the IC50s of all these cell lines is provided in Figure 1E. These data suggest that the acquired resistance to docetaxel results in the cross-resistance to cabazitaxel.

### 3.2. P-gp Was Overexpressed in LNCaP95-DR Cells and Tariquidar Restored Sensitivity to Docetaxel and Cabazitaxel

**Figure 2:**
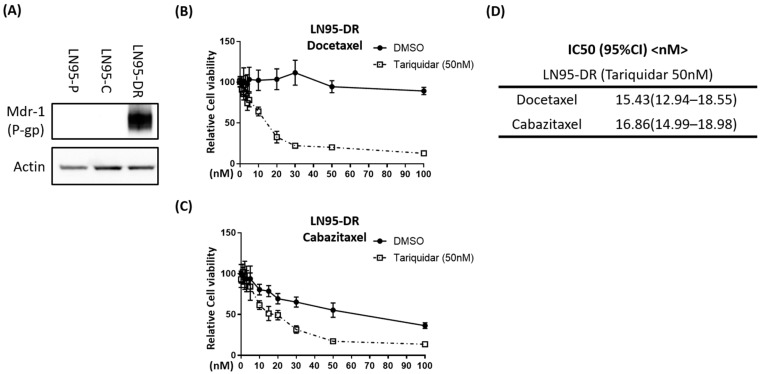
Tariquidar restored the sensitivity of LNCaP95-DR to docetaxel and cabazitaxel. (**A**) Levels of P-gp protein in LN95-P, LN95-C, and LN95-DR cell lysates using b-actin as a loading control; Effects of inhibition of p-gp on the viability of LN95-C and LN95-DR cells incubated with DMSO or a combination of tariquidar (50 nM, inhibitor of P-gp) and increasing concentrations of docetaxel (**B**) or cabazitaxel (**C**); (**D**) Table showing the IC50s of docetaxel and cabazitaxel in LN95-DR cells incubated with a combination of 50 nM tariquidar.

Consistent with a known mechanism of acquired resistance to taxanes, P-gp was overexpressed in LNCaP95-DR cells as measured by the Western blot analysis (Figure 2A). To test whether this high level of P-gp protein in LNCaP95-DR cells played a direct role in the resistance to docetaxel and cabazitaxel, a P-gp inhibitor was tested. Tariquidar is a potent P-gp antagonist that inhibits P-gp mediated drug efflux [30,31,32,33]. We found that the monotherapy with tariquidar showed no effect on the proliferation of LNCaP95-DR (data not shown), whilst tariquidar restored the sensitivity of LNCaP95-DR cells to both docetaxel and cabazitaxel (Figure 2B–D). These data indicated that the cross-resistance between docetaxel and cabazitaxel in LNCaP95-DR cells was mainly mediated by P-gp.

### 3.3. Expression of AR-V7-Regulated Genes Was Increased in LNCaP95-DR

**Figure 3:**
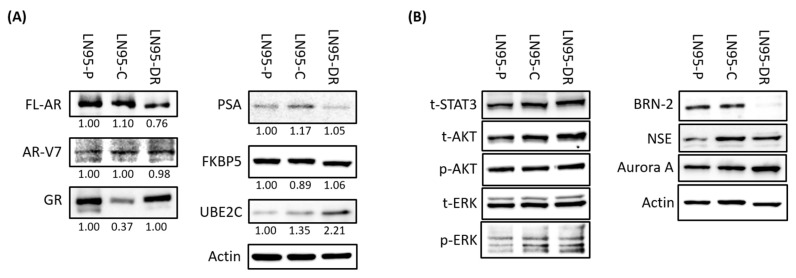
Levels of proteins suspected to play a role in the resistance to therapies for castration-resistant prostate cancer (CRPC). Western blot analyses using whole cell lysates from cell lines. Expression of proteins in androgen receptor (AR) and AR-V7 signaling (**A**), Jak/STAT, PI3K/AKT/mTOR, Ras/MAPK pathway and neuroendocrine markers (**B**). The ratio among the three cell lines was described, which was normalized to that in LN95-P, comparing the protein expression values of FL-AR and AR-V7 regulated molecules normalized to beta-actin as an internal control (**A**).

**Figure A1:**
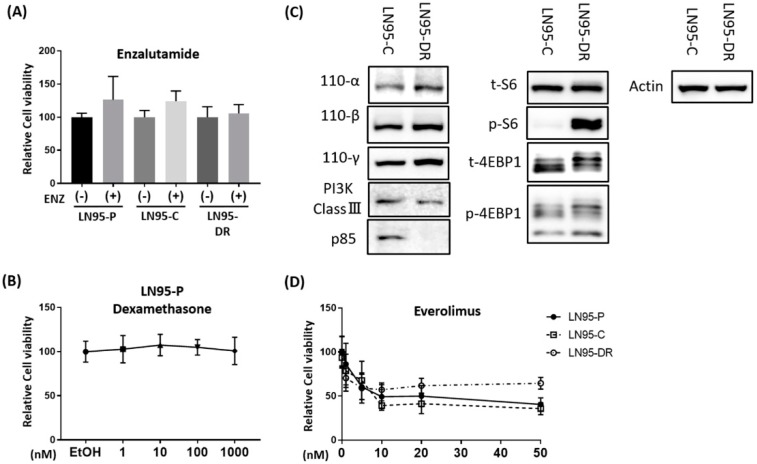
Effect of enzalutamide, dexamethasone, or everolimus on taxane-resistant cells. (**A**) Resistance of LN95-P, LN95-C, and LN95-DR cell viability to 10 μM enzalutamide for 72 h; (**B**) Dose response of dexamethasone on the growth and viability of LN95-P cells; (**C**) Levels of proteins related to the PI3K/Akt/mTOR axis in whole cell lysates; (**D**) Dose response of everolimus on the viability of LN95-P, LN95-C, and LN95-DR cells.

To elucidate other potential contributing factors involved in the mechanism of taxane resistance and provide clues for possible intervention, we compared the levels of expression of several key genes in LNCaP95-DR cells using Western blot analysis and real-time RT-qPCR. LNCaP95-DR cells had higher levels of glucocorticoid receptor (GR), UBE2C, and phosphorylated S6 (pS6), but lower levels of BRN-2 proteins as compared to levels in LNCaP95-C (Figure 3A,B and Figure A1C).

**Figure 4:**
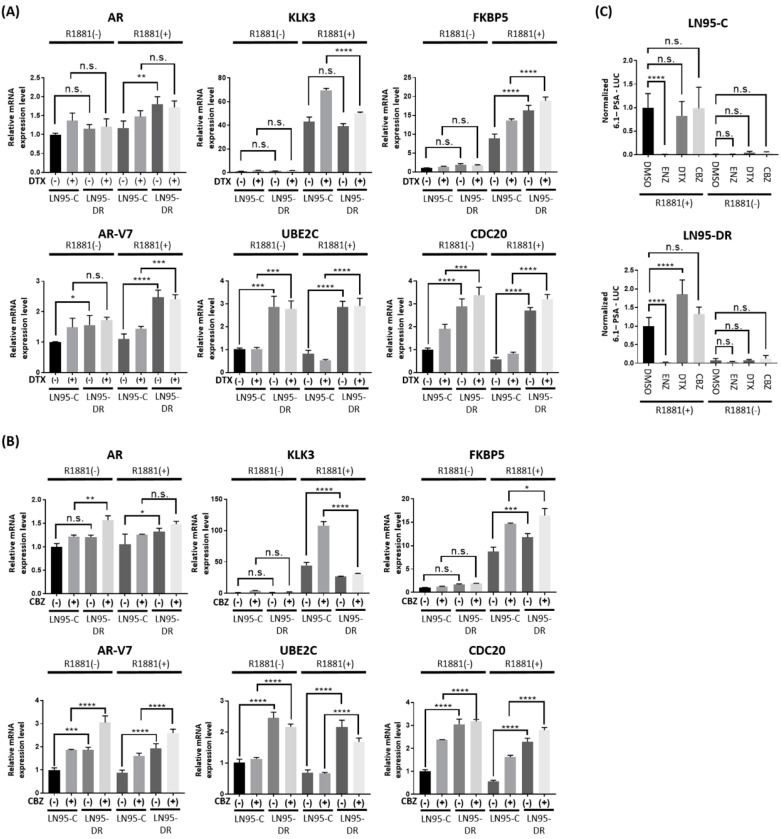
Increased expression of AR-V7 target genes in LNCaP95-DR. Levels of mRNA for FL-AR, AR-V7 and their target genes plus PSA (6.1 kb)-luciferase activities in LN95-C and LN95-DR in response to the synthetic androgen R1881 and taxanes. Transcript levels of FL-AR, KLK3, FKBP5, AR-V7, UBE2C, and CDC20 normalized to transcript levels of GAPDH. LN95-C and LN95-DR were treated with DMSO, docetaxel (5 nM) (**A**) or cabazitaxel (10 nM) (**B**) for 1 h prior to the addition of R1881 (1 nM) or EtOH for 48 h. For the luciferase assay (**C**), LN95-C and LN95-DR were treated with DMSO, enzalutamide (10 µM), docetaxel (5 nM), or cabazitaxel (10 nM) for 1 h prior to treatment with R1881 (1 nM) or EtOH for 48 h. n.s.: not significant; * *p* < 0.05; ** *p* < 0.01; *** *p* < 0.001; **** *p* < 0.0001.

Real-time RT-qPCR revealed that the transcript levels of FL-AR and its target gene KLK3 in LNCaP95-DR cells did not differ from those in LNCaP-C cells, whereas the transcript levels of AR-V7 and its target genes, UBE2C and CDC20, were all increased in LNCaP95-DR cells as compared to levels in LNCaP-C cells (Figure 4). Interestingly, neither docetaxel nor cabazitaxel suppressed the expression of genes regulated by FL-AR (Figure 4A,B). FL-AR was functional in LNCaP95-DR cells as indicated by the induction of PSA-luciferase activity, as well as KLK3 and FKBP5 gene expression in response to the synthetic androgen, R1881 (Figure 4). Neither docetaxel nor cabazitaxel reduced the transcriptional activity of FL-AR in LNCaP95-C or LNCaP95-DR when measuring a PSA-luciferase reporter or endogenous expression of KLK3 and FKBP5 in response to R1881 (Figure 4).

BRN2 is a transcription factor that is proposed to play a role in enzalutamide-induced neuroendocrine transdifferentiation [34]. LNCaP95-DR cells had reduced expression of this transcription factor (Figure 3). Therefore, we tested whether the altered expression of BRN2 might correlate to increased sensitivity to enzalutamide. Unfortunately, no difference in cell viability was measured in response to enzalutamide in LNCaP95-DR cells (Figure A1A). All cell lines remained resistant to enzalutamide.

Glucocorticoid receptor (GR), is proposed to play a role in CRPC as an alternative steroid receptor for AR [35]. Although levels of GR protein were elevated in the LNCaP95 cells (Figure 3), the GR agonist, dexamethasone, did not affect the proliferation of the LNCaP95 cells (Figure A1B). This suggested that the GR is not driving proliferation in this model.

Alterations in expression of components of the PI3K/Akt/mTOR occur in 42% of primary prostate tumors and 100% of metastatic tumors [36]. Thus, targeting the PI3K/Akt/mTOR pathway is considered a promising approach for the treatment of CRPC [15,37,38]. To determine a possible role of this pathway, western blot analysis was performed to assess the status of this pathway in LNCaP95-DR cells. We found no significant change in the expression levels of proteins related to the PI3K/Akt/mTOR pathway, with the exception of pS6 (Figure 3B and Figure A1C). However, the mTOR inhibitor, everolimus, did not mediate differential effects in the different cell lines (Figure A1D), which could suggest that an increased pS6 expression was not important for the acquired resistance to docetaxel.

### 3.4. Knockdown of AR-V7 Has No Effect on Sensitivity to Docetaxel and Cabazitaxel

**Figure 5:**
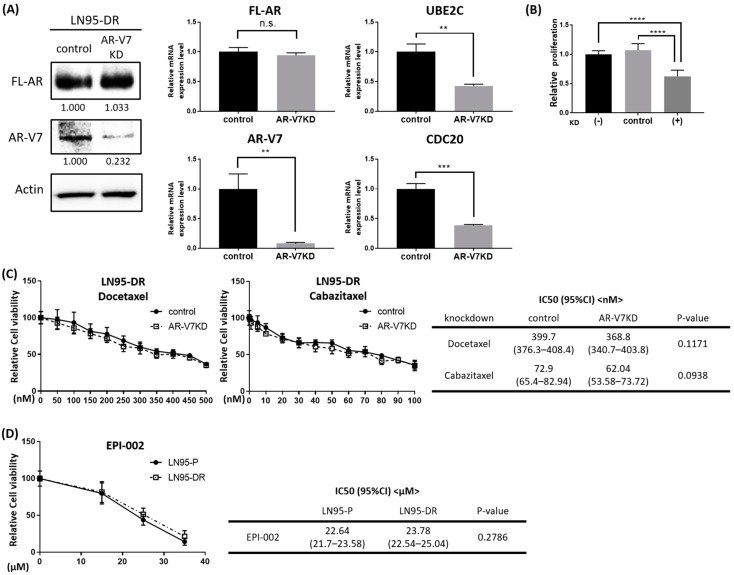
EPI-002 inhibits proliferation of LN95-DR. (**A**) Levels of proteins of FL-AR and AR-V7 and transcripts of FL-AR, AR-V7, UBE2C, and CDC20 in LN95-DR that were transfected with AR-V7 siRNA. After 48 h of transfection with 5 nM AR-V7 siRNA, LN95-DR cells were incubated in serum-free conditions for 48 h prior to collecting the proteins and for 96 h prior to collecting RNA. Transfection with AR-V7 siRNA sufficiently decreased the expression of AR-V7 and the target genes (UBE2C and CDC20) without affecting the level of the FL-AR; (**B**) Knockdown of AR-V7 decreased the proliferation of LN95-DR by 37%. After 48 h of transfection with 5 nM AR-V7 siRNA, LN95-DR cells were incubated in serum-free conditions for 72 h prior to measuring proliferation; (**C**) Dose response curves for docetaxel or cabazitaxel on the viability of LN95-DR cells treated 48 h after transfection with 5 nM AR-V7 siRNA (AR-V7KD). The table shows IC50s of docetaxel and cabazitaxel in LN95-DR cells after knockdown of AR-V7; (**D**) Dose response curve for EPI-002 on proliferation of both LN95-P and LN95-DR cells as measured by the BrdU ELISA assay. The Table shows IC50 values of EPI-002 in LN95-P and LN95-DR cells. n.s.: not significant; ** *p* < 0.01; *** *p* < 0.001; **** *p* < 0.0001.

Given the expression of AR-V7, and its target genes UBE2C and CDC20 being increased in LNCaP95-DR, we examined whether AR-V7 may contribute to the acquisition of resistance to taxanes and whether the targeting of AR-V7 might be a good intervention strategy. To test these we used two approaches, knockdown of AR-V7 and an inhibitor of the AR-Vs transcriptional activities. When AR-V7 expression was transiently knocked down in LNCaP95-DR cells using small interfering RNA (siRNA) (Figure 5A), proliferation of LNCaP95-DR cells was decreased by 37% (Figure 5B). AR-V7 knockdown did not restore the sensitivity of LNCaP95-DR cells to docetaxel or cabazitaxel (Figure 5C). 

AR-NTD targeting drugs are a potential treatment strategy for CRPC represented by LNCaP95-DR cells which have acquired resistance to enzalutamide, docetaxel, and cabazitaxel. This is because AR-NTD is essential for the transcriptional activities exerted by both the FL-AR and AR-Vs. Thus, antagonists of AR-NTD, such as EPI-002, could have a therapeutic effect on LNCaP95 driven by AR-V7. Importantly, EPI-002 had an inhibitory effect on the proliferation of LNCaP95-DR cells that was similar to the effect measured with the parental LNCaP95 cells (Figure 5D). Together, the data revealed that AR-NTD-targeting drugs are a feasible intervention for taxane-resistant prostate cancers that are driven by AR-Vs.

## 4. Discussion

AR-V7 is a major splice variant expressed in human prostate cancer that is associated with the development and progression of CRPC [39,40]. AR-V7 potentially contributes to the resistance to enzalutamide and abiraterone in CRPC [11,41,42]. The involvement of AR-V7 in taxane resistance is not well understood. The LNCaP95 cell line was derived from the LNCaP cell line and it has acquired resistance to androgen depletion conditions. LNCaP95 cells express full-length AR and AR-V7, but the level of AR-V567es is negligible [43]. Proliferation of LNCaP95 cells is driven by AR-V7, despite the endogenous expression of functional FL-AR [39,44]. These cells are resistant to enzalutamide [45]. To elucidate whether AR-V7 plays a role in the acquired resistance to taxanes, the LNCaP95-DR cell line was developed and used as a model for CRPC.

Resistance to taxanes can be associated with the overexpression of P-gp [16,17], which has been confirmed in CRPC patients [18]. Levels of P-gp expression are higher in docetaxel-resistant TaxR and DU145-DTXR cells as compared to that in docetaxel-sensitive, parental C4-2B and DU145 cells, respectively [46,47]. Similarly, P-gp is overexpressed in docetaxel-resistant DU145R and CWR22rv1R cells derived from the parental DU145 and CWR22rv1 cells, respectively [48]. Consistent with these reports, we showed that P-gp was overexpressed in LNCaP95-DR cells, and that tariquidar treatment restored sensitivity to docetaxel. Therefore, overexpression of P-gp played a major role in the acquired resistance of LNCaP95 to docetaxel. Tariquidar is an anthranilic acid-derived third-generation P-gp inhibitor. Its efficacy has been evaluated in several clinical trials on different types of cancer including lung cancer, but there are no reports on its use for prostate cancer. Our study was the first report using tariquidar for prostate cancer cell lines.

Cabazitaxel is a next-generation semisynthetic taxane chemotherapeutic agent that is effective in patients with docetaxel-resistant CRPC [49]. In the TROPIC clinical trial, cabazitaxel significantly improved the overall survival in CRPC patients during or after docetaxel treatment, but the survival benefit was limited to 2.4 months [20]. In our in vitro study, LNCaP95-DR cells were resistant to high doses of docetaxel with an IC50 of >400 nM; however, these cells maintained some sensitivity to cabazitaxel with an IC50 of approximately 70 nM (Figure 1C,D). The data was consistent with that observed clinically with the resistance to docetaxel. Importantly, tariquidar restored sensitivity to docetaxel, as well as to cabazitaxel, thereby indicating that the cross-resistance between docetaxel and cabazitaxel was mediated by the overexpression of P-gp.

Expression of AR-V7 is clinically important and has been proposed for the assessment of which patients should receive inhibitors of the androgen receptor or taxanes [24]. In this study, we showed that the expression of AR-V7-regulated genes was increased in LNCaP95-DR, but that knockdown of AR-V7 did not restore sensitivity to docetaxel and cabazitaxel. These data support the idea that AR-V7 was not involved in taxane resistance. Contrary to these data, Tadani-Mulero M et al. reported that the expression of AR-V7 resulted in taxane resistance in a mouse model of CRPC due to the absence of the AR hinge region, which appears to be critical for microtubule binding [25]. This report compared FL-AR with AR-V567es-expressing LuCaP86.2 tumor xenografts and FL-AR with AR-V7-expressing LuCaP23.1 tumor xenografts. That report concluded that AR-V7, but not AR-V567es, was important for resistance to docetaxel which was not supported by the work presented here in the LNCaP95-DR cells. Consistent with data presented in this study, are those obtained from clinical studies that have shown that the detection of AR-V7 in circulating tumor cells from men with metastatic CRPC was not associated with primary resistance to taxane chemotherapy [23,24,50].

Some recent reports suggest that taxanes can inhibit AR signaling in prostate cancer cells [51,52,53]. However, in our study, neither docetaxel or cabazitaxel decreased the expression of genes regulated by FL-AR nor were there any effects on the transcriptional activity of the AR. Zhu ML and Darshan MS examined the effect of taxanes on the androgen/AR axis using very high concentrations of paclitaxel (1 µM and 100 nM, respectively), which were over the clinically effective range of paclitaxel [54,55]. In the present study, we tested docetaxel and cabazitaxel at the concentrations close to their IC50 (5 nM and 10 nM, respectively), which was clinically feasible [56,57]. Our data suggested that taxane chemotherapy did not affect the androgen/AR axis in LNCaP95-C and LNCaP95-DR when used at the clinically feasible concentration. 

AR-NTD is essential for the transcriptional activities of both FL-AR and AR-Vs. Therefore, AR-NTD-targeting therapy has benefits over the drugs targeting the AR-LBD. EPI-002 targets the NTD of the AR and can block the signaling induced by the FL-AR and AR-Vs [58]. In this study, we showed that the inhibitory effect by EPI-002 on the proliferation of LNCaP95-DR cells was similar to that achieved with the parental LNCaP95 cells. LNCaP95-DR proliferation remained driven by AR-V7, suggesting that AR-NTD could be a therapeutic target for cancers such as LNCaP95-DR, with acquired resistance to taxanes and enzalutamide.

## 5. Conclusions

In summary, we have demonstrated that docetaxel-resistant LNCaP95 cells are cross-resistant to cabazitaxel. We showed that resistance to docetaxel and cabazitaxel depended on the increased expression of P-gp and the inhibition of P-gp with tariquidar restored to docetaxel and cabazitaxel. Furthermore, expression of AR-V7-regulated genes was increased in LNCaP95-DR cells, although AR-V7 did not contribute to taxane resistance. Finally, EPI-002, an antagonist of AR-NTD, inhibited proliferation of LNCaP95-DR. In conclusion, the present study described a potential option for the treatment of docetaxel-resistant, AR-V7-driven CRPC.
